# A multi-access identification key based on colour patterns in ladybirds (Coleoptera, Coccinellidae)

**DOI:** 10.3897/zookeys.758.22171

**Published:** 2018-05-14

**Authors:** Séverin Jouveau, Mathilde Delaunay, Régine Vignes-Lebbe, Romain Nattier

**Affiliations:** 1 Institut Systématique Evolution Biodiversité (ISYEB), Muséum national d’Histoire naturelle, CNRS, Sorbonne Université, EPHE, 57 rue Cuvier, CP 50, 75005 Paris, France; 2 INRA - UMR 1202 Biodiversité Gènes & Communautés, 69 route d’Arcachon, 33612 Cestas, France

**Keywords:** Citizen science, Coccinellidae, Coccinellini, Chilocorini, Epilachnini, France, interactive identification keys, ladybirds, Xper

## Abstract

An identification key based on French ladybird colouration is proposed for the tribes Chilocorini, Coccinellini, and Epilachnini. These tribes were chosen based on their relatively limited species diversity, as well as their large size and high colour diversity, making them easy to observe and collect. The identification key runs on Xper^3^ software, which allows the building of structured knowledge bases and online free-access keys. The online interactive Xper key is available at http://french-ladybird.identificationkey.fr.

## Introduction

The identification of species is central in ecology, conservation biology, systematics, and related disciplines (species inventories and community studies, ecosystem management, establishment and improvement of environmental public policies, taxonomic reviews, and management of natural history collections) (Oliver 1988, Hebert et al. 2003, Smith et al. 2008, Vander Zanden et al. 2010). Europe is one of the best-known parts in the world in terms of biodiversity (Fontaine et al. 2012), especially concerning distribution patterns at the country scale. This has been highlighted by the release of the Fauna Europaea database since 2004 (de Jong et al. 2014), which gathers the scientific names and distributions of all living European animal species and is assembled by a large network of specialists. However, most new species are described by non-professional taxonomists (Fontaine et al. 2012) and the distribution of a majority of organisms remains poorly known. Citizen science programs aim to fill that gap (Silvertown 2009), thanks to the participation of amateurs and the general public to the inventory and description of life (e.g., National Biodiversity Network in the UK, Swedish Species Gateway in Sweden, Chicago Wilderness Project in the USA, Vigie-Nature in France; see Silvertown 2009). From this perspective, visual and interactive identification of species offers tremendous potential for the general public.

If the identification of large and charismatic animals may be easy, the majority of organisms require expert skills for accurate identification and the inability to identify species represents a major challenge known as the Taxonomic Impediment (SCBD 2010). The most basic requirement for people studying and working on biodiversity aspects is the availability of species identification guides. However, easy-to-use identification guides for non-taxonomists and the general public are scarce and available for relatively few taxonomic groups (SCBD 2010). Consequently, the other features of organisms (such as distribution, ecology, biology) remain poorly known (Costello et al. 2006, SCBD 2010).

Coccinellidae is a family of beetles popular and appreciated by naturalists and the general public. Because these animals have ecological and economic values as predators of pest insects (e.g. aphids, scale insects), their identification may be of importance for naturalists, amateurs and professionals (Hemptinne et al. 2005, Hodek and Honěk 2009, Ali et al. 2014). Several citizen science programs aim to describe the distribution patterns of this group: we can mention for instance the Harlequin Ladybird Survey (http://www.harlequin-survey.org) and the Ladybird Survey (http://www.ladybird-survey.org) in the UK, the Lost Ladybug Project (http://www.lostladybug.org) and the Buckeye Lady Beetle Blitz (https://entomology.osu.edu/about-us/multimedia/buckeye-lady-beetle-blitz) in the US, and the Coccinula Recording Scheme in Belgium (Baugnée et al. 2011). The data collected led to a significant number of scientific works that have been published (e.g., Brown et al. 2008, Comont et al. 2012, 2014, Gardiner et al. 2012, Purse et al. 2015).

Single-access identification keys consist of a series of identification steps that form a single and unique identification path for a given taxon. Although it is a very powerful tool for identifying species, the user cannot choose the character to be observed (the answer for every single step must be known), and the identification is impossible if some characters are missing (e.g., if the specimen is poorly preserved). Moreover, this type of keys cannot be modulated or adapted to various kinds of publics, environmental conditions, season, or geographical location.

Most North American or European ladybird identification keys are single-access and difficult to use for non-specialists (Dauguet 1949, Iablokoff-Khnzorian 1982, Gordon 1985, Chapin and Brou 1991). Others are mainly based on shape and colour, but most characters need specific vocabulary, which makes the key still too complicated for the general public in the perspective of citizen science programs (Belgium: Baugnée and Branquart 2000; West of France: Le Monnier and Livory 2003; British Isles: Roy et al. 2013; North of France: Declercq et al. 2014).

Modern tools developed along with digital technologies and data processing make identification easier for the user. In this perspective, several interactive identification keys (IIK) are available online (e.g., http://www.ladybird-survey.org/bbc/spotter.php, http://www.discoverlife.org/20/q?guide=Ladybug), but most of them are only digital versions of single-access keys and maintain the same difficulties for the user.

A multi-access interactive key is a computer-aided identification tool that makes it possible to find correct names of species where the user enters attributes (character-state values) of the specimen (Dallwitz et al. 2013). The advantages compared to conventional keys are as follows: characters can be used in any order, characters are ordered to start with the one that best separates the remaining taxa, keys can be completed with illustrations (pictures, drawings) and texts explaining the terminology used, correct identifications can be obtained despite errors made by the user (FloraBase – https://florabase.dpaw.wa.gov.au/keys; Dallwitz et al. 2013). The software also includes the possibility to print a single-access key for field identification if needed, and to weight characters according to the user skills and abilities (students, general public, naturalists…). Despite the advantages provided by multi-access interactive keys, none has been produced for ladybirds so far.

This study aims to i) release the first multi-access digital interactive identification key for French ladybirds based on colour that takes into account intraspecific variability; and ii) study and discuss the discriminating power of the characters: can we identify species by colour pattern only? What are the most discriminating characters?

## Materials and methods

### Taxonomic coverage

As the aim of the key is to provide an identification tool for the general public in the perspective of citizen science programs, we have restricted the taxonomic coverage to the tribes Chilocorini, Coccinellini and Epilachnini (Table 1). Members of these tribes are relatively large (3–9 mm) and display a great diversity of colours, making them easily detectable in their environment and identifiable by non-specialists. We also included the most common colour forms, trying to cover most of the intraspecific variability of these species.

**Table 1.** Taxonomic coverage of the study.

**Coccinellinae Latreille, 1807**	Chilocorini Mulsant, 1846	*Chilocorus bipustulatus* Linnaeus, 1758
		*Chilocorus renipustulatus* Scriba, 1791
		*Exochomus octosignatus* Gebler, 1830
		*Exochomus quadripustulatus* Linnaeus, 1758
		*Parexochomus nigromaculatus* Goeze, 1777
	Coccinellini Latreille, 1807	*Halyzia sedecimguttata* Linnaeus, 1758
		*Psyllobora vigintiduopunctata* Linnaeus, 1758
		*Vibidia duodecimguttata* Poda, 1761
		*Anisosticta novemdecimpunctata* Linneaus, 1758
		*Coccinula quatuordecimpustulata* Linnaeus, 1758
		*Coccinula sinuatoinarginata* Faldermann, 1837
		Tytthaspis sedecimpunctata Linnaeus 1761 – f. duodecimpunctata
		Adalia bipunctata Linnaeus, 1758 – f. annulata
		Adalia bipunctata Linnaeus, 1758 – f. pantherina
		Adalia bipunctata Linnaeus, 1758 – f. quadrimaculata
		Adalia bipunctata Linnaeus, 1758 – f. sexpustulata
		Adalia bipunctata Linnaeus, 1758 – f. typica
		Adalia conglomerata Linnaeus, 1758 – f. decas
		Adalia decempunctata Linnaeus, 1758 – f. decempustulata
		Adalia decempunctata Linnaeus, 1758 – f. guttatopunctata
		Adalia decempunctata Linnaeus, 1758 – f. lutea
		Adalia decempunctata Linnaeus, 1758 – f. quadripunctata
		Adalia decempunctata Linnaeus, 1758 – f. scribai
		Adalia decempunctata Linnaeus, 1758 – f. subpunctata
		Adalia decempunctata Linnaeus, 1758 – f. terna
		Adalia decempunctata Linnaeus, 1758 – f. typica
		*Anatis ocellata* Linnaeus, 1758
		Aphidecta obliterata Linnaeus, 1758 – f. typica
		*Calvia decemguttata* Linnaeus, 1767
		*Calvia quatuordecimguttata* Linnaeus, 1758
		*Calvia quindecimguttata* Fabricius, 1777
		*Ceratomegilla alpina* Villa A. & Villa G. B., 1835
		*Ceratomegilla notata* Laicharting, 1781
		*Ceratomegilla rufocincta* Mulsant, 1850
		*Ceratomegilla undecimnotata* Schneider D.H. 1792
		*Coccinella venusta* Weise, 1879
		*Coccinella hieroglyphica* Linnaeus, 1758
		*Coccinella magnifica* Redtenbacher, 1843
		*Coccinella quinquepunctata* Linnaeus, 1758
		*Coccinella septempunctata* Linnaeus, 1758
		*Coccinella undecimpunctata* Linnaeus, 1758
		Harmonia axyridis Pallas, 1773 – f. conspicua
		Harmonia axyridis Pallas, 1773 – f. novemdecimsignata
		Harmonia axyridis Pallas, 1773 – f. spectabilis
		*Harmonia conformis* Boisduval, 1835
		Harmonia quadripunctata Pontoppidan, 1763 – f. sedecimpunctata
		Harmonia quadripunctata Pontoppidan, 1763 – f. typica
		Hippodamia septemmaculata DeGeer, 1775 – f. cestiva
		*Hippodamia tredecimpunctata* Linnaeus, 1758
		Hippodamia variegata Goeze, 1777 – f. quinquemaculata
		Hippodamia variegata Goeze, 1777 – f. undecimpunctata
		Hippodamia variegata Goeze, 1777 – f. carpini
		Hippodamia variegata Goeze, 1777 – f. constellata
**Coccinellinae Latreille, 1807**	Coccinellini Latreille, 1807	Hippodamia variegata Goeze, 1777 – f. velox
		*Myrrha octodecimguttata* Linnaeus, 1758
		*Myzia oblongoguttata* Linnaeus, 1758
		*Oenopia conglobata* Linnaeus, 1758
		*Oenopia doublieri* Mulsant, 1846
		*Oenopia impustulata* Linnaeus, 1767
		*Oenopia lyncea* Olivier, 1808
		Propylea quatuordecimpunctata Linnaeus, 1758 – f. weisei
		*Sospita vigintiguttata* Linnaeus, 1758
	Epilachnini Chevrolat in Dejean, 1837	*Henosepilachna argus* Geoffroy in Fourcroy, 1785
		*Henosepilachna elaterii* Rossi, 1794
		Subcoccinella vigintiquatuorpunctata Linnaeus, 1758 – f. limbata
		Subcoccinella vigintiquatuorpunctata Linnaeus, 1758 – f. typica

The current taxonomy (Seago et al. 2011) and the species list follow Tronquet (2014) and include native, introduced, and acclimated species. Sixty-six taxa are included in the key (47 species and 19 intraspecies colour forms). Several species were removed from this list: introduced and non-acclimated species (*Chilocorus kuwanae* Silvestri, 1909; *C. nigritus* Fabricius, 1798; *C. stigma* Say, 1835; *Hippodamia convergens* Guérin-Méneville, 1842; *Olla v-nigrum* Mulsant, 1866), or doubtful records (*Anisosticta strigata* Thünberg, 1795; *Cynegetis impunctata* Linnaeus, 1767). *Platynaspis luteorubra* Goeze, 1777 (Chilocorinae, Platynaspini) was also removed due to its small size (2.5–3.5mm). Since only a few discriminating characters are known that are not reliable with colour patterns, *Henosepilchna angusticollis* Reiche, 1862 is not discriminated from its congener *H. argus* Geoffroy in Fourcroy, 1785 in the key.

Specimens were examined in the collection of the Muséum national d’Histoire naturelle, Paris, France (**MNHN**) and their data are available at https://science.mnhn.fr/institution/mnhn/collection/ec/search.

### Characters used in the key

A list of 21 morphological characters based on colour and shape is defined, mainly from existing identification keys (Iablokoff-Khnzorian 1982, Baugnée and Branquart 2000, Roy et al. 2013, Declercq et al. 2014) (Table 2). Only characters that are visible to the naked eye or with a ×10 hand lens are included. The character nomenclature follows Roy et al. (2013), except for characters #10, 11, 15, 16, 17, and 18. All characters were treated as discrete.

**Table 2.** List of descriptors used in this study.

**Pronotum**
1. Pronotum colours
2. Pattern on pronotum
3. Number of pronotum patterns
4. Type of pronotum patterns
**Elytra**
5. Elytra main colour (background)
6. Elytra markings
7. Colour of elytra markings
8. Number of elytra markings
9. Type of elytra markings
10. Number of lateral lines in the elytra markings
11. Number of longitudinal lines in the elytra markings
12. A spot in the first third of the elytra
13. One of the spots reaches the rim of the elytra
14. Cream ring around dots
15. Dark sutural elytra band
16. Scutellar spot
17. Shape of the scutellar spot
18. White marks between the scutellar spot and the elytra basis
19. Distinct rim around the edge of the elytra
20. Elytra covered with short hairs
**Underside**
21. Small white triangular marks on the underside below both the middle and front legs

### Interactive identification key construction and statistics

Digitalization of the 47 species was performed using Xper^2^ v.2.3.2 (Ung et al. 2010) and transferred to Xper^3^ (Vignes-Lebbe et al. 2016). These softwares are dedicated to manage structured taxonomic descriptions, to analyse these descriptions and to produce keys (Kerner et al. 2011, Corvez and Grand 2014, Martin et al. 2015). A wiki and a documentation of Xper^3^ are available at http://wiki.xper3.fr/index.php/UserManualXper3.

**Figure 1.**
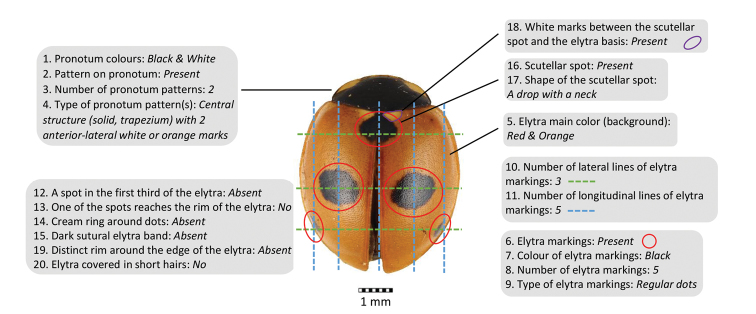
Description of *Coccinella quinquepunctata* following the list of terms used in this study.

An Xper knowledge base is a set of items described using the same model and terminology, and documented by texts and images. In this key there are 66 items covering 47 species and 19 intraspecies colour forms. The descriptive model consists of a hierarchy of descriptors and a chosen terminology for expressing different possible values (states). The descriptors are the 21 morphological characters previously described. Some of them are consistent only if some conditions are true for another descriptor and these dependencies define a hierarchical structure of descriptors (Table 2). The complete terminology (descriptors and states) is documented by images and texts in order to avoid misinterpretation, a crucial point for relevant identifications with the key. Figure 1 presents the description of the species *Coccinella quinquepunctata* following these model and terms.

Xper^3^ was also used to compare species and genera. For each descriptor, the comparison tests are able to distinguish a pair of items. Three different measures are available (Burguière et al. 2013). The result is displayed as a table with different colours to separate three cases: (1) items have the same values for a given descriptor (= no discrimination), (2) one pair of items is completely distinct for a given descriptor (= total discrimination), (3) at least one pair of items has not equal values for the descriptor, but these values overlap (= partial discrimination). For a given descriptor the sum of the comparison for all pairs of items is a measure of its ability to distinct taxa (discriminatory power).

The discriminatory power, which represents the quantitative assessments of the ability of a descriptor to distinguish taxa, is measured with the Xper original index (Lebbe 1991) implemented in the Xper^2^ software. This index is based on the incompatibility between descriptions. Two taxa are incompatible (or dissimilar or discriminated) if for one given descriptor there are no states of descriptors in common. For each descriptor the index value ranges between 0 (null discriminatory power of the descriptor) and 1 (the descriptor can distinguish all taxa).

Comparisons within and between genera are made with the “compare groups” and “compare items” options of Xper^3^. For the comparison between genera, we estimated the number of discriminating characters, weighted or not by the number of colour forms. A subset of descriptors sufficient to discriminate the total of descriptors with the same efficiency was also calculated with the “minset” tool (Lebbe and Vignes 1992, Ziani et al. 1994).

### Comparison with standard keys

Two types of keys are available: free-access keys and single-access keys (Hagedorn et al. 2010). A free-access key is a very flexible identification key allowing the user to choose the characters he or she wants to describe. Another web service (Ikey+) (Burguière et al. 2013) is a single-access key builder. A single-access key is a classical key in which descriptors are ordered steps. The topology of the key is a tree and it is possible to compute some indices on the tree: number of maximal steps, length of the paths, etc.

A single-access identification key was generated by IKey+ under Xper^3^ with the default option and the Xper score method. In this case we show four statistics by taxon: the number of steps, the length of the shortest and the longest paths, and the average length of paths. This key was then compared with five single-access keys for European ladybirds (Dauguet 1949, Baugnée and Branquart 2000, Le Monnier and Livory 2003, Roy et al. 2013, Declercq et al. 2014).

## Results

### Structure and analysis of the key

The consistency of the knowledge base has been tested with the “Checkbase” functionality of Xper^3^: no items share the same description and all items are described. The base is 100% complete. Twenty-one descriptors are used: five do not have any dependence (either father or son), four are parent descriptors (for which two are also child descriptors) and 14 are child descriptors (for which two are also parent descriptors). Ninety-eight states are described (minimal/maximal/average number of states: 2/12/4.67).

### Discriminatory power of descriptors (Table 3)

**Table 3.** List of the discriminating power efficiency of descriptors. Those written in bold are sufficient for discriminating all taxa.

Category	Descriptor	XPER index	Number of descriptor states
**Pronotum**	**4. Type of pronotum pattern(s)**	**0.85**	**10**
**Elytra**	**8. Number of elytra markings**	**0.85**	**12**
Elytra	10. Number of lateral lines of elytra markings	0.83	8
**Elytra**	**11. Number of longitudinal lines of elytra markings**	**0.82**	**7**
**Pronotum**	**3. Number of pronotum patterns**	**0.71**	**9**
Elytra	17. Shape of the scutellar spot	0.68	4
**Elytra**	**5. Elytra main colour (background)**	**0.53**	**7**
Elytra	7. Colour of elytra markings	0.52	**6**
**Elytra**	**13. One of the spots reaches the rim of the elytra**	**0.49**	**2**
Elytra	18. White marks between the scutellary spot and the elytra basis	0.47	2
Elytra	16. Scutellar spot	0.46	2
**Elytra**	**12. A spot in the first third of the elytra**	**0.4**	**2**
**Elytra**	**9. Type of elytra markings**	**0.32**	**6**
**Elytra**	**15. Dark sutural elytra band**	**0.18**	**2**
Elytra	19. Distinct rim around the edge of the elytra	0.14	2
**Elytra**	**6. Elytra markings**	**0.12**	**2**
Elytra	20. Elytra covered in short hairs	0.12	2
Pronotum	2. Pattern on pronotum	0.11	2
Pronotum	1. Pronotum colours	0.09	7
Elytra	14. Cream ring around dots	0.06	2
**Underside**	**21. Small white triangular marks on the underside below both the middle and front legs**	**0.03**	**2**

The four most discriminating characters (XPER index >0.8) are the type of pronotum patterns (#5), the number of elytra markings (#8), and the number of lateral (#10) and longitudinal (#11) lines of elytra markings. These characters can separate taxa in 7 to 13 groups. For example, the two most discriminating characters (#5 and #8) split all the remaining taxa in 10–13 different groups including 2–13 taxa per group.

The characters #14 and #21 are the least discriminating as they both have an XPER index below 0.8. These characters are binary and split all taxa in two unequal groups (60 vs 2 for the character #14, 65 vs 1 for the character #21). Despite its weak discriminating power, the character #21 is the only one that can distinguish the two species *Coccinella septempunctata* and *C. magnifica*. Eleven descriptors are sufficient to separate all taxa (Table 3, in bold).

### Comparison within and between genera

**Comparison within a genus: Coccinella (Table 4)**

**Table 4.** Comparison within genus: *Coccinella* (six species), showing informative characters (in bold) and constant characters (in regular). The intersection column shows what is constant within the genus.

	*Coccinella venusta*	*Coccinella hieroglyphica*	*Coccinella magnifica*	*Coccinella quinquepunctata*	*Coccinella septempunctata*	*Coccinella undecimpunctata*	UNION	INTERSECTION
1. Pronotum colours	Black; White							
**2. Elytra main colour (background)**	Black; Orange	Black; Orange	Red; Orange	Red; Orange	Red; Orange	Red; Orange	Black; Orange; Red	Orange
3. Pattern on pronotum	Present							
4. Number of pronotum patterns	2							
5. Type of pronotum patterns	Central structure (solid, trapezium) with 2 anterior-lateral white or orange marks							
6. Elytra markings	Present							
**7. Colour of elytra markings**	Red; Orange; Black	Black	Black	Black	Black	Black	Red; Orange; Black	Black
**8. Number of elytra markings**	1; 2	7	7	5	7	10–14	1; 2; 5; 7; 10–14	
**9. Type of elytra markings**	Other	Ovoid shape spot; Other	Regular dots	Regular dots	Regular dots	Regular dots	Ovoid shape spot; Regular dots; Other	
**10. Number of lateral lines of elytra markings**	not applicable	3	3	3	3	3	3	
**11. Number of longitudinal lines of elytra markings**	not applicable	4	5	5	5	5	4; 5	
**12. Scutellar spot**	Absent	Absent	Present	Present	Present	Present	Absent; Present	
13. Distinct rim around the edge of the elytra	absent							
14. Elytra covered in short hairs	No							
**15. Small white triangular marks on the underside below both the middle and front legs**	Absent	Absent	Present	Absent	Absent	Absent	Absent; Present	
16. Cream ring around dots	Absent							
17. Dark sutural elytra band	Absent							
**18. Shape of the scutellar spot**	not applicable	not applicable	A drop (spot with a neck)	A drop (spot with a neck)	A drop (spot with a neck)	A drop (spot with a neck)	A drop (spot with a neck)	
**19. White marks between the scutellar spot and the basis of elytra**	not applicable	not applicable	Present	Present	Present	Present	Present	
**20. One of the spots reaches the rim of the elytra**	No	Yes	No	No	No	No	Yes; No	
**21. A spot in the first third of the elytra**	Absent	Present	Present	Absent	Present	Present	Absent; Present	

Among the 21 characters, 12 are informative (in blue) whereas the other nine are constant and cannot discriminate within this genus (in red). The intersection column shows what is constant in *Coccinella*, therefore helping with the description of the genus: black and white pronotum with two patterns (Central structure - solid, trapezium with two anterior-lateral white or orange marks), elytra with different markings, but always devoid of rim around the edge, short down hairs, cream rings around dots, or dark sutural band.

**Comparison between the 24 genera included in the study (Table 5)**

**Table 5.** Comparison between the 24 genera included in the study, showing the most constant or variable genus, weighted or not by the intraspecific variability taken into account in this study (number of colour forms).

	Number of species studied	Number of colour forms studied	Number of discriminating characters within genus	
			with all colours forms	weighted by the number of colour forms
***Adalia***	3	14	16 (76%)	1.1
***Calvia***	3	3	5 (23%)	1.7
***Ceratomegilla***	4	4	13 (62%)	3.3
***Chilocorus***	2	2	4 (19%)	2
***Coccinella***	6	6	11 (52%)	1.8
***Coccinula***	2	2	1 (5%)	0.5
***Exochomus***	2	2	7 (33%)	3.5
***Harmonia***	3	6	14 (67%)	2.3
***Henosepilachna***	2	2	3 (14%)	1.5
***Hippodamia***	3	7	10 (48 %)	1.4
***Oenopia***	4	4	10 (48%)	2.5
***Subcoccinella***	1	2	–	–
***Anatis***	1	1	–	–
***Anisosticta***	1	1	–	–
***Aphidecta***	1	1	–	–
***Halyzia***	1	1	–	–
***Myrrha***	1	1	–	–
***Myzia***	1	1	–	–
***Parexochomus***	1	1	–	–
***Propylea***	1	1	–	–
***Psyllobora***	1	1	–	–
***Sospita***	1	1	–	–
***Tytthaspis***	1	1	–	–
***Vibidia***	1	1	–	–

Among the genera with at least two species studied, the most constant are *Coccinula* (5% of discriminating characters), *Henosepilachna* (14%) and *Chilocorus* (19%); the most variable are *Adalia* (76%) and *Harmonia* (67%). If weighted by the number of described colour forms per genera, the most constant are still *Coccinula* and *Henosepilachna*; whereas the most variable genera are *Ceratomegilla* and *Exochomus*.

**Single-access identification key and comparison with standard keys**

**Figure 2.**
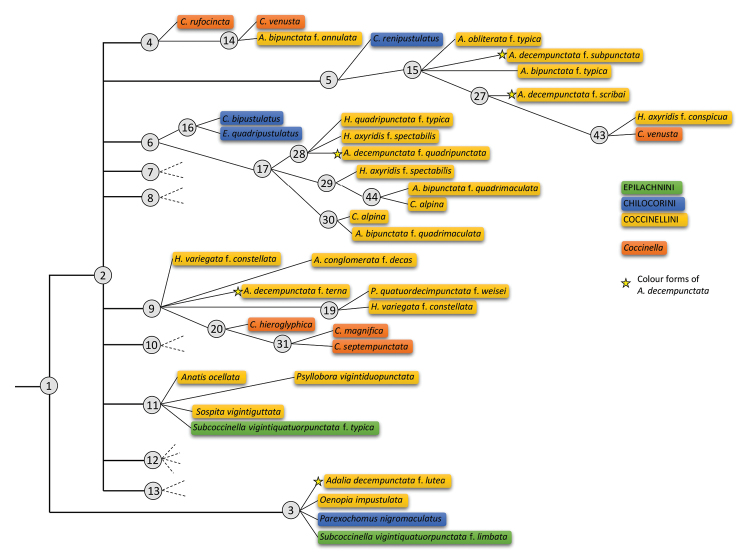
Representation of a part of the single-access identification key generated by IKey+ under Xper^3^ and the Xper score method (statistics detailed in Appendix 1). The taxonomy is highlighted (the three tribes included in this study, the genus *Coccinella* and the colour forms of *Adalia decempunctata*). Numbers in the circles represent the number of steps in the generated key.

For each identification, the descriptive statistics of the generated key (Appendix 1) are: mean 4.2 steps (2–7), 1–4 paths leading to a taxon (mean 1.5). Unlike many other single-access keys, lots of steps for identifying a taxon do not follow the taxonomy. This is the case in the three tribes: for instance, the user can follow five different paths for identifying an Epilachnini species (in green). The same reasoning applies to *Coccinella* species (in red) with six different paths, and the colour forms of *A. decempunctata* (marked with a yellow star) with six different paths (Figure 2).

In comparison to other standard keys (Table 6), this newly generated key is more efficient for finding the taxon, despite its highest number of included species, except for *Coccinula quatuordecimpustulata* and the key of Dauguet (1949). For example, only five steps are required in the generated key for identifying *Coccinella septempunctata*, whereas 8–14 steps are needed in the other keys.

**Table 6.** Comparison between five single-access keys from bibliography and the generated key by IKey+ under Xper^3^ for 13 ladybird species: average number of steps (number of paths, length of the shortest and the longest paths).

	Generated Key - this study 47 species	Dauguet (1949) 35 species	Baugnée and Branquart (2000) 37 species	Le Monnier and Livory (2003) 35 species	Roy et al. (2013) 26 species	Derolez et al. (2014) 35 species
Adalia bipunctata f. typica	**4** (1)	**5** (1)	**8** (1)	**11** (1)	**7** (1)	**9** (1)
*Anatis ocellata*	**3** (2)	**6** (1)	**4.6** (3, 3–7)	**4** (1)	**8** (3, 6–10)	**8** (2, 7–9)
*Anisosticta novemdecimpunctata*	**3** (1)	**5** (1)	**9** (1)	**5** (1)	**10** (1)	**5.5** (2, 3–8)
*Calvia quatuordecimguttata*	**5** (1)	**11** (1)	**12** (1)	**10** (1)	**8** (1)	**11** (1)
*Chilocorus bipustulatus*	**4** (1)	-	**7** (1)	**4** (1)	**4** (1)	**8** (1)
*Coccinella hieroglyphica*	**4** (2)	**10** (1)	**6.5** (4, 4–9)	-	**8** (1)	**8** (2, 7–9)
*Coccinella quinquepunctata*	**3** (1)	**12** (1)	**8** (1)	-	**10** (1)	**13** (1)
*Coccinella septempunctata*	**5** (1)	**13** (1)	**8** (1)	**12** (1)	**11** (1)	**14** (1)
*Coccinella undecimpunctata*	**5** (1)	**11** (1)	**8.5** (2, 8–9)	**11** (1)	**10** (1)	**10.5** (2, 10–11)
*Coccinula quatuordecimpustulata*	**6** (2)	**10** (1)	**5** (1)	**14** (1)	-	**11** (1)
*Myzia oblongoguttata*	**5.5** (2, 5–6)	**6** (1)	**6** (1)	**6** (1)	**7** (1)	**7** (1)
*Oenopia conglobata*	**4** (1)	**10** (1)	**11** (1)	**9** (1)	-	**6** (1)
*Psyllobora vigintiduopunctata*	**3** (1)	**8** (1)	**11** (1)	**12** (1)	**12** (1)	**7** (1)

## Discussion

The work presented in this study led to the release of the first multi-access interactive digital identification key for French ladybirds. The adaptability and great number of possibilities provided by this new generation tool are unparalleled for this group, and make the key very flexible and abundantly illustrated and described, thanks to images and texts. Since it is available online and open to experts for modification, the identification key can easily be improved. It will be possible to add ladybird taxa and to extend the geographic area (e.g., a key to all European ladybirds).

Most classical and single-access keys share characters that are quite difficult to observe for students, naturalists and the general public (e.g. for ladybirds in Dauguet 1949 or Roy et al. 2013: mandibles, tooth on tibia, tarsal claws, mesosternal epimera, abdominal post-coxal lines). Here, all taxa are distinguishable with only 11 characters focusing on markings (number and shape). All characters used in this new key are visible to the naked eye or with a x10 hand lens; therefore this tool is designed for non-specialists. Using this key, most species can be identified through pictures only, as it is already the case in the identification key for the photographic survey of flower visitors (Spipoll citizen science program, www.spipoll.org), also built with Xper^3^.

Identification in the field is traditionally realised with paper-printed keys, but recent developments of mobile devices make it possible to use portable version of digital keys. In this perspective, the project “KeyToNature” (www.keytonature.eu) aims to develop new, more convenient and paper-free identification tools, for use within schools and universities across Europe and available on a variety of mobile platforms (laptops, smartphones). However these keys are single access, e.g., MobileKey (Weber and Hagedorn 2010) or the iRecord Ladybirds mobile application, and do not support all the possibilities provided by the free-access keys. Recently, a set of software and applications for transferring the information present in a local Xper database to an Android application has been developed (Troudet 2012). For example, the Malaco-fr application provides an interactive way to identify French snails in the field and without internet connection (Gargominy and Ripken 2011). The free-access key of French ladybirds described in this study will soon be transferred to such a mobile application, which will make it possible to use this tool in educative programs such as “Vigie-nature école” (https://www.vigienature-ecole.fr).

Both experienced and inexperienced users are likely to succeed in identifying problematic species (Morse et al. 1996, Drinkwater 2009) if they use convenient, multi-access interactive digital keys. The general public can discriminate species and several colour forms only with colour/form characters and a ×10 lens. Moreover, Xper^3^ provides possibilities for collaborative work and editing through its website. All the online features make it possible to update the knowledge base easily (e.g. adding numerous colour forms or acclimated species, or new characters such as ecological data). This kind of key aims to increase the curiosity of the general public, and to collect more data on the biology and distribution of species.

## Appendix 1

**Table:**

STATISTICS				
Taxon	Number of paths leading to taxon	Length of the shortest path leading to taxon	Average length of paths leading to taxon	Length of the longest path leading to taxon
Adalia bipunctata f. annulata	1	4	4	4
Adalia bipunctata f. pantherina	1	4	4	4
Adalia bipunctata f. quadrimaculata	2	5	5.5	6
Adalia bipunctata f. sexpustulata	2	3	3.5	4
Adalia bipunctata f. typica	1	4	4	4
Adalia conglomerata f. decas	1	3	3	3
Adalia decempunctata f. decempustulata	2	7	7	7
Adalia decempunctata f. guttatopunctata	4	7	7	7
Adalia decempunctata f. lutea	1	2	2	2
Adalia decempunctata f. quadripunctata	1	5	5	5
Adalia decempunctata f. scribai	1	5	5	5
Adalia decempunctata f. subpunctata	1	4	4	4
Adalia decempunctata f. terna	1	3	3	3
Adalia decempunctata f. typica	2	6	6	6
*Anatis ocellata*	2	3	3	3
*Anisosticta novemdecimpunctata*	1	3	3	3
Aphidecta obliterata f. typica	2	2	3	4
*Calvia decemguttata*	2	6	6	6
*Calvia quatuordecimguttata*	1	5	5	5
*Calvia quindecimguttata*	2	6	6	6
*Ceratomegilla alpina*	2	5	5.5	6
*Ceratomegilla notata*	2	6	7	8
*Ceratomegilla rufocincta*	1	3	3	3
*Ceratomegilla undecimnotata*	1	8	8	8
*Chilocorus bipustulatus*	1	4	4	4
*Chilocorus renipustulatus*	1	3	3	3
*Coccinella hieroglyphica*	2	4	4	4
*Coccinella magnifica*	1	5	5	5
*Coccinella quinquepunctata*	1	3	3	3
*Coccinella septempunctata*	1	5	5	5
*Coccinella undecimpunctata*	1	5	5	5
*Coccinella venusta*	2	4	5	6
*Coccinula quatuordecimpustulata*	2	6	6	6
*Coccinula sinuatomarginata*	1	5	5	5
*Exochomus octosignatus*	1	2	2	2
*Exochomus quadripustulatus*	1	4	4	4
*Halyzia sedecimguttata*	1	5	5	5
Harmonia axyridis f. conspicua	2	6	6	6
Harmonia axyridis f. novemdecimsignata	2	4	4.5	5
Harmonia axyridis f. spectabilis	2	5	5	5
*Harmonia conformis*	3	3	4	5
Harmonia quadripunctata f. sedecimpunctata	2	3	3	3
Harmonia quadripunctata f. typica	1	5	5	5
*Henosepilachna argus*	2	4	5	6
*Henosepilachna elaterii*	1	4	4	4
Hippodamia septemmaculata f. cestiva	2	3	3.5	4
*Hippodamia tredecimpunctata*	1	4	4	4
Hippodamia variegata f. undecimpunctata	2	5	6	7
Hippodamia variegata f. quinquemaculata	1	3	3	3
Hippodamia variegata f. carpini	1	4	4	4
Hippodamia variegata f. constellata	2	3	3.5	4
Hippodamia variegata f. velox	2	5	5.5	6
*Myrrha octodecimguttata*	1	5	5	5
*Myzia oblongoguttata*	4	5	5.5	6
*Oenopia conglobata*	1	4	4	4
*Oenopia doublieri*	1	4	4	4
*Oenopia impustulata*	1	2	2	2
*Oenopia lyncea*	4	5	5.5	6
*Parexochomus nigromaculatus*	1	2	2	2
Propylea quatuordecimpunctata f. weisei	2	4	4	4
*Psyllobora vigintiduopunctata*	1	3	3	3
*Sospita vigintiguttata*	1	3	3	3
Subcoccinella vigintiquatuorpunctata f. limbata	1	2	2	2
Subcoccinella vigintiquatuorpunctata f. typica	1	3	3	3
Tytthaspis sedecimpunctata f. duodecimpunctata	2	5	5.5	6
*Vibidia duodecimguttata*	1	5	5	5
**AVERAGE**	**1.53**	**4.197**	**4.364**	**4.53**
